# Identification of prognostic genes in adrenocortical carcinoma microenvironment based on bioinformatic methods

**DOI:** 10.1002/cam4.2774

**Published:** 2019-12-19

**Authors:** Xiao Li, Yang Gao, Zicheng Xu, Zheng Zhang, Yuxiao Zheng, Feng Qi

**Affiliations:** ^1^ Department of Urology Jiangsu Cancer Hospital & Jiangsu Institute of Cancer Research & Affiliated Cancer Hospital of Nanjing Medical University Nanjing China; ^2^ Department of Radiology Jiangsu Cancer Hospital & Jiangsu Institute of Cancer Research & Affiliated Cancer Hospital of Nanjing Medical University Nanjing China; ^3^ Hepato‐Pancreato‐Biliary Center Zhongda Hospital School of Medicine Southeast University Nanjing China; ^4^ Department of Urology The First Affiliated Hospital of Nanjing Medical University Nanjing China

## Abstract

**Background:**

To identify prognostic genes which were associated with adrenocortical carcinoma (ACC) tumor microenvironment (TME).

**Methods and materials:**

Transcriptome profiles and clinical data of ACC samples were collected from The Cancer Genome Atlas (TCGA) database. We use ESTIMATE (estimation of stromal and Immune cells in malignant tumor tissues using expression data) algorithm to calculate immune scores, stromal scores and estimate scores. Heatmap and volcano plots were applied for differential analysis. Venn plots were used for intersect genes selection. We used protein‐protein interaction (PPI) networks and functional analysis to explore underlying pathways. After performing stepwise regression method and multivariate Cox analysis, we finally screened hub genes associated with ACC TME. We calculated risk scores (RS) for ACC cases based on multivariate Cox results and evaluated the prognostic value of RS shown by receiver operating characteristic curve (ROC). We investigated the association between hub genes with immune infiltrates supported by algorithm from online TIMER database.

**Results:**

Gene expression profiles and clinical data were downloaded from TCGA. Lower immune scores were observed in disease with distant metastasis (DM) and locoregional recurrence (LR) than other cases (*P* = .0204). Kaplan‐Meier analysis revealed that lower immune scores were significantly associated with poor overall survival (OS) (*P* = .0495). We screened 1649 differentially expressed genes (DEGs) and 1521 DEGs based on immune scores and stromal scores, respectively. Venn plots helped us find 1122 intersect genes. After analysing by cytoHubba from Cytoscape software, 18 hub genes were found. We calculated RS and ROC showed significantly predictive accuracy (area under curve (AUC) = 0.887). ACC patients with higher RS had worse survival outcomes (*P* < .0001). Results from TIMER (tumor immune estimation resource) database revealed that HLA‐DOA was significantly related with immune cells infiltration.

**Conclusion:**

We screened a list of TME‐related genes which predict poor survival outcomes in ACC patients from TCGA database.

## Graphical abstract

To identify prognostic genes in adrenocortical carcinoma (ACC) tumor microenvironment (TME).

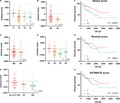

## 1 INTRODUCTION

Adrenal tumors are very common, approximately 3%‐10% of the human population suffers from the disease, and most of them are nonfunctional adrenocortical adenomas (benign lesions).^1^ However, adrenocortical carcinoma (ACC) is extremely rare with an incidence of 0.7‐2 cases per million people annually.^2^, ^3^, ^4^ Surgery is the first‐line therapy for patients with localized ACC (stage I, II diseases and most of stage III diseases), while different therapy methods (molecularly targeted therapy, immunotherapy, adjuvant therapy, and so on) or multidisciplinary approaches are applied in advanced patients in order to achieve improvement in the prognosis.^5^ The prognosis of patients with ACC is poor because most of them are present with metastatic or advanced diseases, in which surgical resection is not amenable. Additionally, about two‐thirds of localized patients suffer recurrence and systemic therapy is always required.^6^, ^7^, ^8^ Hence, tumor burden is one of the most important prognostic factors in ACC patients. As reported, 5‐year survival rate in patients with stage I disease was about 80% while only 13% in patients with stage IV diseases.^9^

Tumor microenvironment (TME) includes not only the tumor cells themselves, but also the surrounding fibroblasts, immune and inflammatory cells, glial cells and other cells, as well as the intercellular substance, microvessels, and biomolecules infiltrated in the nearby area.^10^, ^11^, ^12^ Nowadays, numerous studies focused on the relationship between TME and survival outcomes or recurrence. Ren et al reported that interaction between stromal cells and epithelial/cancer cells affected the cancer progress in pancreatic cancer.^13^ Jian et al^14^ discovered that higher TH1 was an important predictor of poor prognosis in patients with nonsmall cell lung cancer (NSCLC). However, studies on the role of TME in prognosis in adrenal tumors were few and most of them focused on adrenocortical adenomas. Kitawaki et al^15^ firstly histologically investigated the TME of autonomous hormone‐secreting adrenocortical adenomas. He demonstrated that immune cell infiltration uniquely existed in cortisol‐producing adrenocortical adenomas and immune cells enter the tumor site through the CXCL12‐CXCR4 signaling and eliminate the aging tumor cells.

With the advent of the era of big data, transcriptome profiles and clinical data of cancer patients can be easily obtained from some public database such as The Cancer Genome Atlas (TCGA) database, which includes abundant cancer‐causing genomic alterations data of various malignancies,^16^ and many algorithms were created to explore the tumor purity based on the TCGA database.^17^, ^18^ Additionally, Estimation of Stromal and Immune cells in Malignant Tumours using Expression data (ESTIMATE), a novel algorithm proposed by Yoshihara et al^17^ was utilized to calculate the fraction of immune and stromal cells in malignancy. ESTIMATE algorithm generates stromal score (that captures the presence of stroma in tumor tissue), immune score (that represents the infiltration of immune cells in tumor tissue), and estimate score (that infers tumor purity and equal in number to stromal score and immune score). Previous studies had reported that scores calculated by ESTIMATE algorithm could be utilized as a predictor of nontumor cell infiltration in TMEs of colon cancer, prostate cancer, and breast cancer.^19^, ^20^, ^21^ However, no research discussed the TME of ACC using ESTIMATE algorithm. Hence, we conducted this study to identify prognostic genes which were associated with ACC TME.

## 2 METHODS AND MATERIALS

### 2.1 Clinical characteristics and ESTIMATE data collection

Level 3 gene transcriptome profiles were obtained from The Cancer Genome Atlas (TCGA) database (https://portal.gdc.cancer.gov/) and 79 ACC patients was enrolled in our study. RNA expression for ACC Multiforme was collected using IlluminaHiSeq (version: 2017‐10‐13). The following corresponding clinical characteristics were extracted: sex, age, cancer type, status, topography, lymph node and metastasis (TNM) based on American Joint Committee on Cancer (AJCC), pathological stage and new event of the disease. Then, we download the ACC survival outcomes through the Genomic Data Commons (GDC) tool from TCGA portal. Normalization process was performed by utilizing package limma.^22^ Immune scores, stromal scores, and estimate scores were calculated by applying ESTIMATE algorithm which R script was downloaded from the website (https://sourceforge.net/projects/estimateproject/). The statistical results were shown in Table S1.

### 2.2 Survival analysis and correlation analysis

Unpaired t test was used to analysis the relationship between immune scores, stromal scores, estimate scores and AJCC‐N and AJCC‐M. Ordinary one‐way Analysis of Variance (ANOVA) was used to analysis the relationship between immune scores, stromal scores, estimate scores and AJCC‐T, pathological stage, new event of the disease. *P* < .05 was considered as statistically significant. Kaplan‐Meier (K‐M) analysis which based on the immune scores, stromal scores, and estimate scores of ACC patients was performed through survival package.^23^, ^24^ In K‐M analysis we used dichotomous variable. Log‐rank test was performed and *P < *.05 represented a statistically significant result.

### 2.3 Heatmaps and clustering analysis

Enrolled samples were divided as high‐ and low‐level groups according to the median of three scores from the ESTIMATE method. In immune score groups, the transcriptome data were analyzed by limma package under the standard of | log(FC) | > 1 and False Discovery Rate (FDR) < 0.05. After that, we used pheatmap package and clustering analysis to explore the differential high and low expression genes among the different immune score levels. We used volcano plot performed by ggplot2 package to exhibit significantly differentially expressed genes. The cut |log_2_FC| was 1 and cut *P* value was .05. Besides, we performed the same analysis procedure in two stromal score groups. In this way, we obtained intersect genes among immune scores and stromal scores. The result was exhibited by VennDiagram package.^25^

### 2.4 Construction of PPI network, GO analysis, KEGG, and GSEA

The protein‐protein interaction (PPI) network was constructed from (search tool for recurring instances of neighbouring genes) STRING database^26^ and the result was visualized with Cytoscape software (version 3.7.1).^27^ The inside‐software plugin cytoHubba was helped to identified hub genes among interested genes with 12 different topological analysis methods.^28^ We calculated the network nodes and excluded the networks less than 10 nodes. After that, online tool DAVID (https://david.ncifcrf.gov/) was exploited to construct the gene ontology (GO) analysis.^29^ What's more, Kyoto Encyclopedia of Genes and Genomes (KEGG) analysis was performed for pathway analysis using org.Hs.eg.db package, clusterProfiler, org.Hs.eg.db, enrichplot, and ggplot2 packages. Bar charts were used to reveal the pathway annotation and enrichment. Top 20 pathway enrichment was analyzed and shown by bubble chart. The *q*‐value < 0.05 was considered as significant. The results of Cellular Component (CC), Molecular Function (MF), and Biological Process (BP) were all collected and shown as barplot. We set “c2.cp.kegg.v6.2.symbols.gmt gene sets” as gene set database, “Illumina_Human.chip” as chip platform when setting parameters, FDR < 0.25, |enriched score| > 0.35, and gene size ≥ 35 in Gene Set Enrichment Analysis (GESA) software.^30^

### 2.5 Overall survival curve and risk score

K‐M plots were generated to select prognostic hub genes. In K‐M analysis we used a dichotomous variable. We used *P < *.05 of log‐rank test to evaluate the relationship. Besides, survival package and “for cycle” R script was performed to select prognostic hub genes. Then, Multivariate Cox regression analysis was used to evaluate the risk score in the formula of risk score = Ʃ (*β_i_* × Exp*_i_*) (*i* = the number of prognostic hub genes). After calculating risk scores of ACC patients, we separated all patients in high‐ and low‐risk groups according to the median score. The survival ROC package was used to conduct the receiver operating characteristic curve (ROC).^31^ In addition, we used K‐M plot to visually present the differences in survival outcome between high‐risk and low‐risk groups. In K‐M analysis we used dichotomous variable.

### 2.6 Timer database analysis

To explore the specific association of hub genes with immune cells, we used the deconvolution algorithm provided by the TIMER (tumor immune estimation resource) database (https://cistrome.shinyapps.io/timer/) for analysis. The immune cells involved in the analysis include: B cell, CD4^＋^ T cell, CD8^＋^ T cell, macrophage, neutrophil, dendritic cell. In addition, the association between tumor purity and hub genes was also analyzed.

### 2.7 Statistical analysis

Survival package was applied in multivariate Cox regression analysis and K‐M analysis. Limma package was applied in differential analysis. Unpaired t test and ordinary one‐way Analysis of Variance (ANOVA) were used to analysis data from two or more groups. Statistical analysis was implemented on R software (version 3.5.2). When we used FDR to evaluate the results, FDR < 0.05 indicated that the results were statistically significant. In addition, when we used *P* to evaluate the results, *P* < .05 indicated that the results were statistically significant.

## 3 RESULTS

### 3.1 Lower immune scores significantly associated with malignant progression of ACC

**Figure 1:**
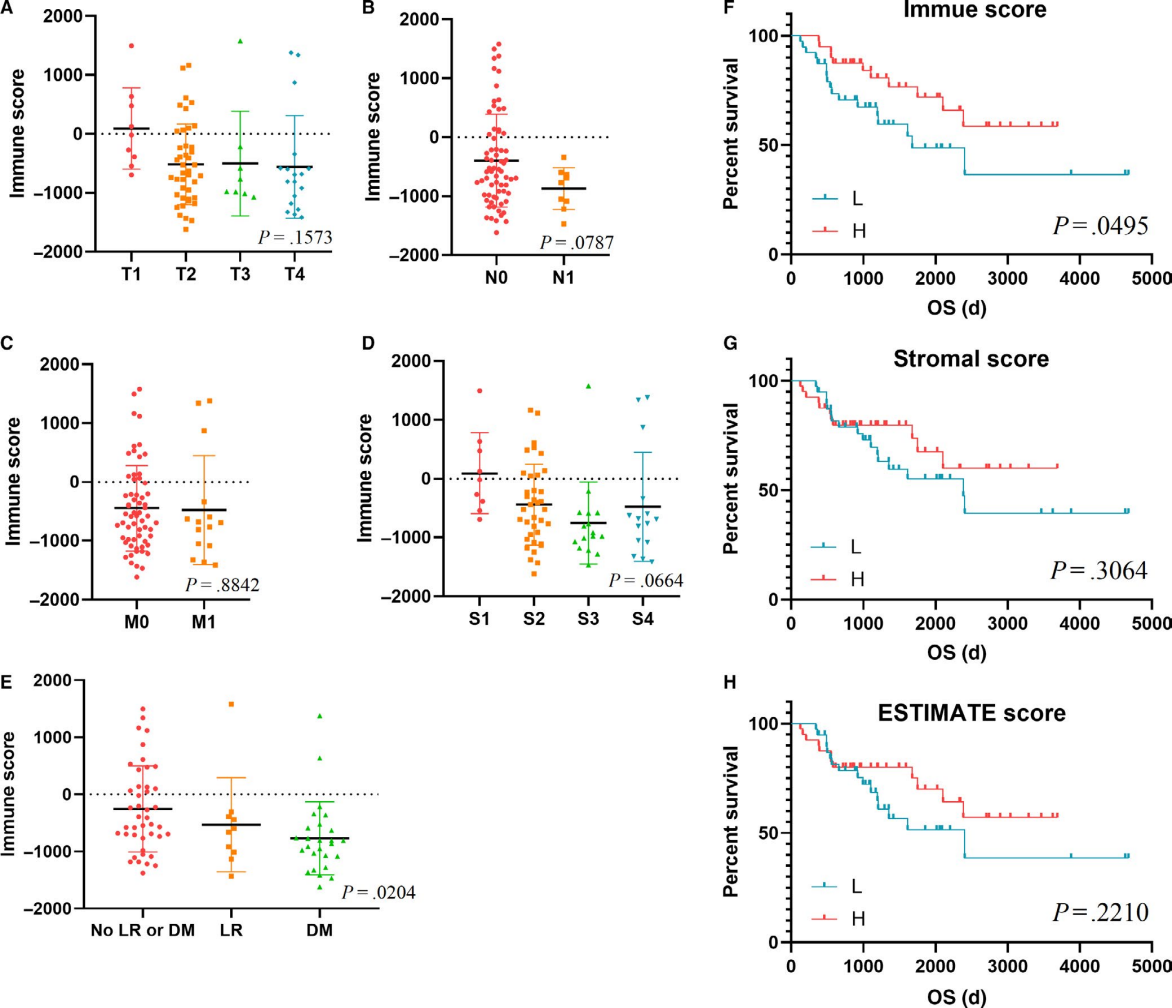
Lower immune scores significantly associated with malignant progression of ACC Scatter plots revealed the distribution of immune scores in AJCC‐T (*P* = 0.1573) (A), AJCC‐N (*P* = 0.0787) (B), AJCC‐M (*P* = 0.8842) (C), tumor stage (*P* = 0.0664) (D) and event of malignant progression (*P* = 0.0204) (E). Lower immune scores were associated with DM and LR than other cases. K‐M survival curve shown that ACC patients with lower immune scores were associated with poor survival outcomes (*P* = 0.0495) (F). Results of K‐M analysis in stromal score (G) and ESTIMATE score (H) shown no statistical significance (*P* = 0.3064, *P* = 0.2210, respectively). DM, distant metastasis; K‐M, Kaplan‐Meier; LR, locoregional recurrence

We downloaded gene expression profiles data of 79 samples from TCGA, including 31 male cases (39.2%) and 48 female cases (60.8%). Selected clinical characteristics were shown in Table 1. Patients’ immune scores (range from −1181.25 to 1577.33), stromal scores (range from −1766.65 to 1519.45), and estimate scores (range from −2947.9 to 3096.78) were listed in Table S1. We explored differences of immune scores according to different clinical characteristics and presented the results in Figure 1A‐E. Lower immune scores were observed in disease with distant metastasis (DM) and locoregional recurrence (LR) than other cases (*P* = .0204). Besides, difference analysis of stromal scores and estimate scores in data groups of clinical characteristics were shown in Figure S1.

**Table 1:** Clinical characteristics of 92 ACC patients included in study from TCGA cohort

	Male (%)	Female (%)	Total (%)
Sex	31(39.2%)	48(60.8%)	79(100%)
Age (years)	48.74 ± 15.67	45.38 ± 15.69	46.70 ± 15.67
Cancer type			
Usual type	29(36.7%)	46(58.2%)	75(94.9%)
Oncocytic type	2(2.5%)	1(1.3%)	3(3.8%)
Myxoid type	0(0.0%)	1(1.3%)	1(1.3%)
Status			
Dead	11(13.9%)	17(21.5%)	28(35.4%)
Alive	20(25.3%)	31(39.2%)	51(64.6%)
T*			
1	3(3.8%)	6(7.6%)	9(11.4%)
2	15(19.0%)	27(34.2)	42(53.2%)
3	4(5.0%)	4(5.0%)	8(10.1%)
4	7(8.9%)	11(13.9%)	18(22.8%)
N*			
0	27(34.2%)	41(51.9%)	68(86.1%)
1	2(2.5%)	7(8.9%)	9(11.4%)
M*			
0	24(30.4%)	38(48.1%)	62(78.5%)
1	5(6.3%)	10(12.7%)	15(19.0%)
Stage*			
I	3(3.8%)	6(7.6%)	9(11.4)
II	15(19.0%)	22(27.8%)	37(46.8%)
III	6(7.6%)	10(12.7%)	16(20.3%)
IV	5(6.3%)	10(12.7%)	15(19.0%)
New event			
Distant metastasis	6(7.6%)	20(25.3%)	26(32.9%)
Locoregional recurrence	5(6.3%)	5(6.3%)	10(12.7%)
New primary tumor	1(1.3%)	0(0.0%)	1(1.3%)
No new event	19(24.1%)	23(29.1%)	42(53.2%)

* There existed a partial deletion of this clinical variable when it was collected.

Furthermore, K‐M analysis revealed that lower immune scores was significantly associated with poor overall survival (OS) (Figure 1F, *P* = .0495). However, difference in survival data was not observed in stromal scores and estimate scores (Figure 1G‐H, *P* = .3064 and *P* = .2210, respectively).

### 3.2 Comparison of gene expression profiles with immune scores and stromal scores

**Figure 2:**
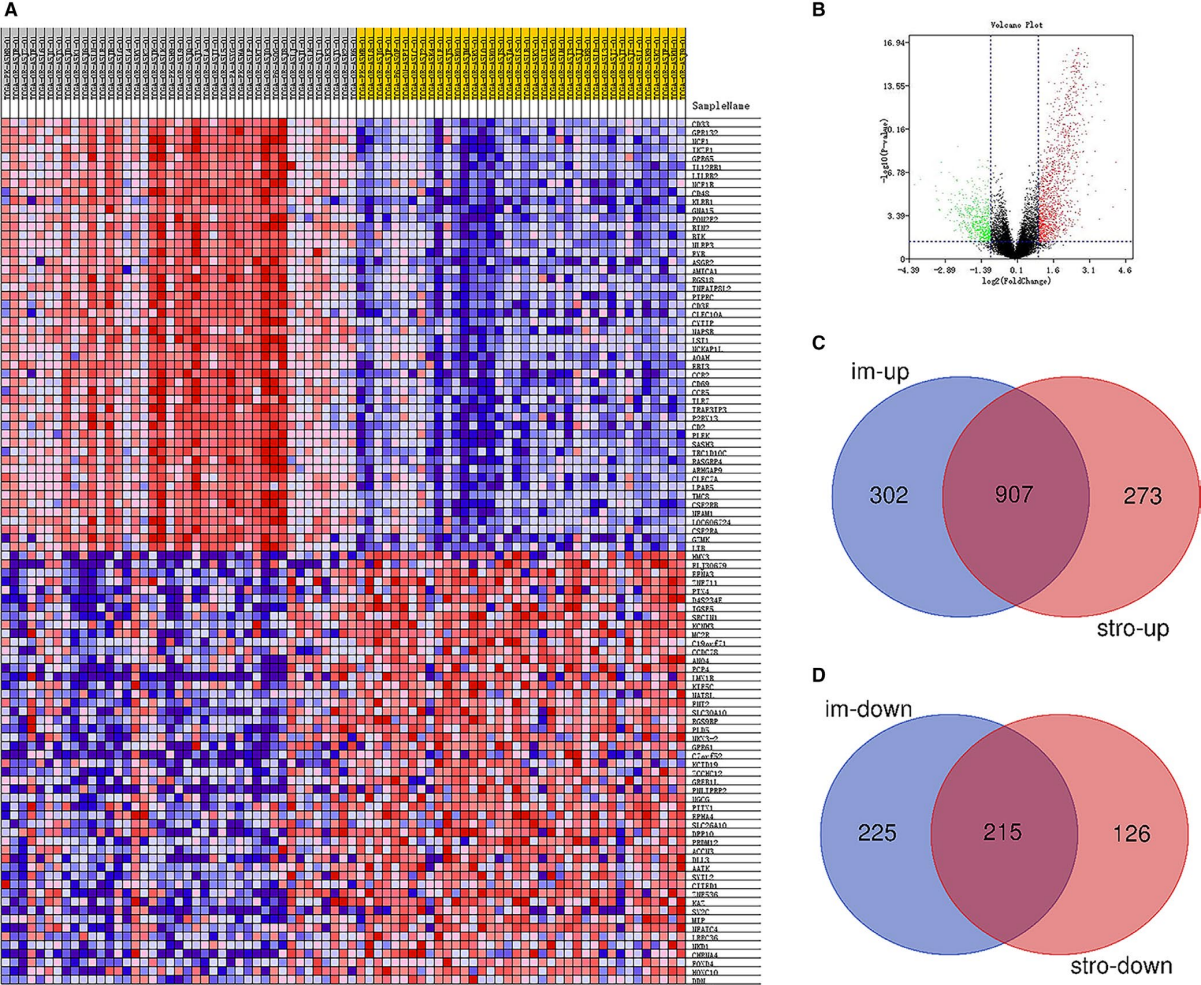
Comparison of gene expression profiles with immune scores and stromal scores. Based on immune scores, clustering analysis was shown in Heatmap (A). Volcano plot exhibit significantly differentially expressed genes (B). Venn plots revealed 1122 intersect genes which including 907 upregulated genes in higher immune/stromal scores (C) and 215 down‐regulated genes in lowerimmune/stromal scores (D)

We used limma package to analyze RNA‐seq data. ACC patients were divided into low‐level group (n = 39) and high‐level group (n = 40) in accordance with median immune scores. Clustering analysis was shown in Heatmap in Figure 2A. Volcano plot which exhibits significantly differentially expressed genes was shown in Figure 2B. Based on immune scores, 1649 differentially expressed genes (DEGs) which contained 1209 upregulated DEGs and 440 downregulated DEGs were screened (|log2fold‐change| > 1, FDR < 0.05, Table S2). Besides, 1521 DEGs which contained 1180 upregulated DEGs and 341 downregulated DEGs were screened based on stromal sores (|log2fold‐change| > 1, FDR < 0.05, Table S3). The heatmap and volcano plot based on stromal scores were shown in Figure S2 A‐B. Venn plots were applied to screen 1122 intersect genes which including 907 upregulated genes in higher immune/stromal scores and 215 downregulated genes in lower immune/stromal scores (Figure 2C,D).

### 3.3 PPI, GO analysis, KEGG analysis, and GSEA

**Figure 3:**
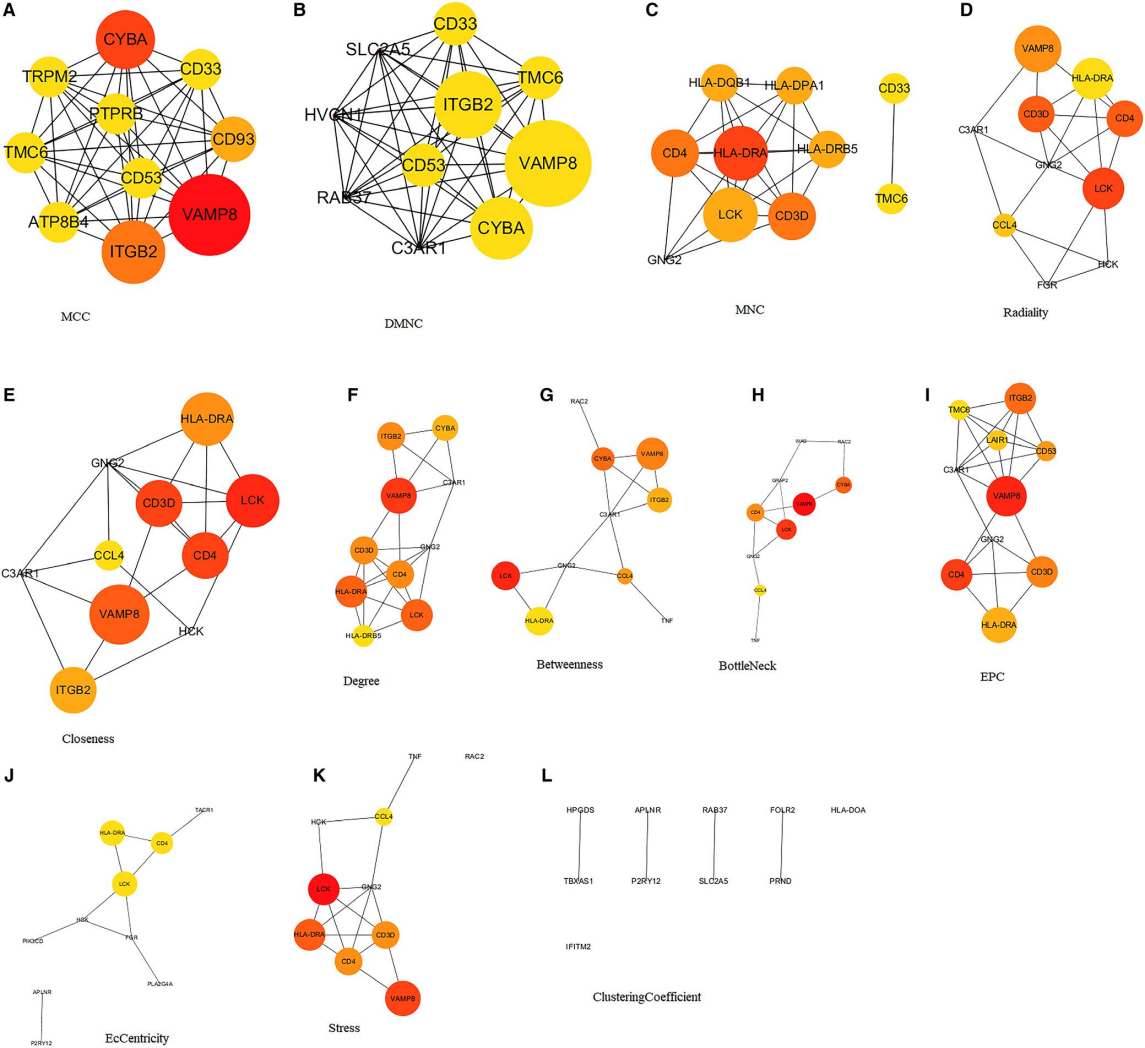
Hub genes screened by 12 algorithms from cytoHubba. The network shown the analysis results generated by the algorithms of MCC (A), DMNC (B), MNC (C), Radiality (D), Closeness (E), Degree (F), Betweenness (G), BottleNeck (H), EPC (I), EcCentricity (J), Stress (K) and ClusteringCoefficent (L). The size of the circle indicates the node degree

**Figure 4:**
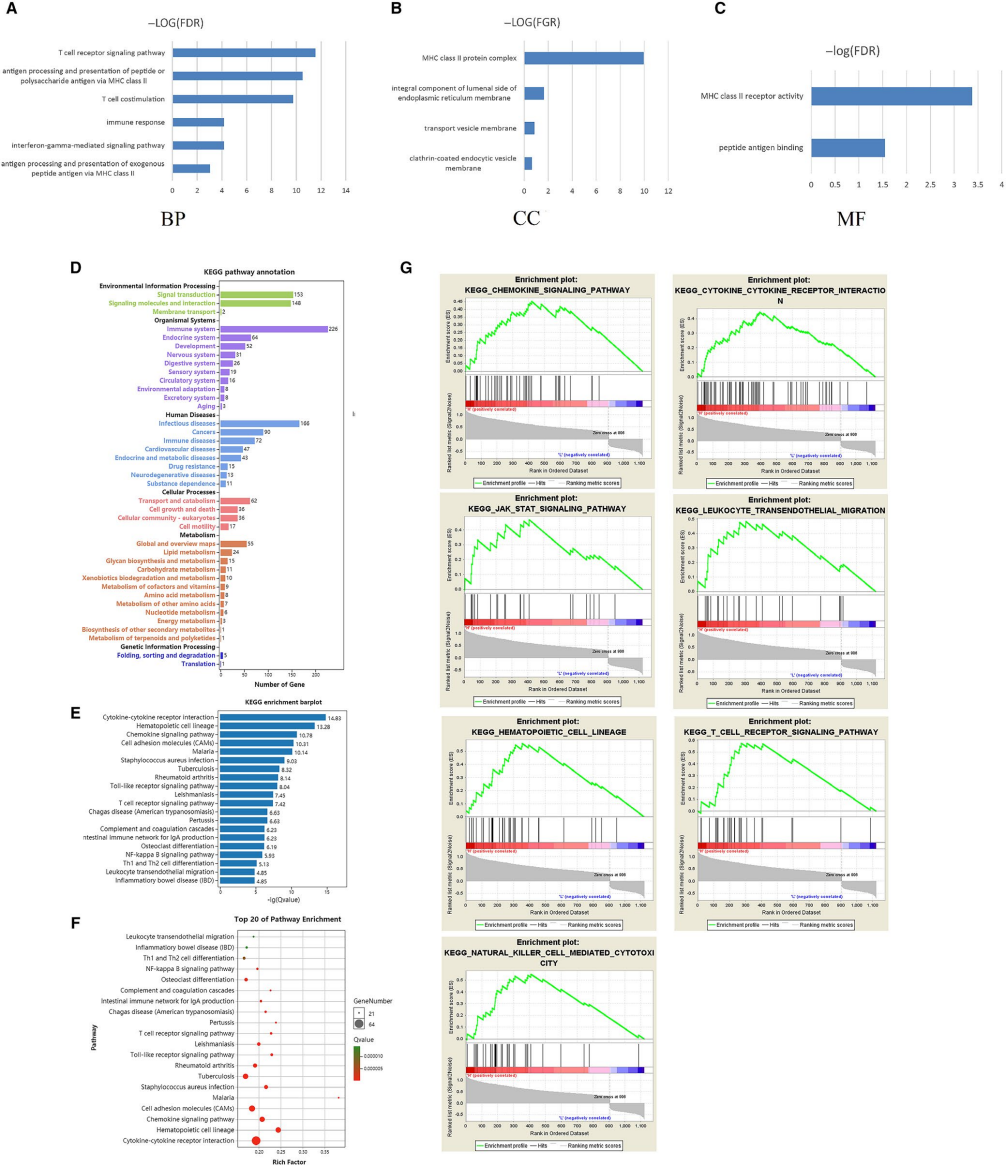
GO analysis, KEGG analysis and GSEA. Results of BP (A), CC (B), and MF (C) in GO analysis revealed the relationship between hub genes and functional pathways. Pathway annotation (D) and enrichment (E) were revealed by bar charts and top 20 pathway enrichment was shown by bubble chart (F) in KEGG analysis. GSEA analysis (G) was performed to further screen the significant pathway between higher immune scores group and lower immune scores group. The *q*‐value＜0.05 was considered as significance. BP, biological process; CC, cellular component; GO, geneontology; GSEA, Gene Set Enrichment Analysis; KEGG, Kyoto Encyclopedia of Genes and Genomes; MF, molecular function

About 1122 intersect genes were uploaded and analyzed by online SRING tool (https://string-db.org/, minimum required interaction score: 0.9) and Cytoscape software. Then, 18 hub genes (CD4, HLA‐DRA, HCK, CD53, RAC2, HLA‐DRB5, RAB37, CD93, FOLR2, GRAP2, HLA‐DOA, HLA‐DPA1, HPGDS, LAIR1, PTPRB, TACR1, TBXAS1, WAS) were selected from the result analyzed by cytoHubba. The network generated by the 12 topological analysis methods was shown in Figure 3. The GO analysis revealed that hub genes might be associated with T‐cell receptor signaling pathway, antigen processing and presentation of exogenous peptide antigen via MHC class II, T‐cell costimulation, MHC class II protein complex, and MHC class II receptor activity (Figure 4A‐C). In addition, KEGG pathway annotation and enrichment were analyzed and shown in Figure 4D‐E. Top 20 pathway enrichment was revealed by bubble chart in Figure 4F.

GSEA analysis was performed to further screen the significant pathway between higher immune scores group and lower immune scores group. We selected seven immune‐related pathways: hematopoietic cell lineage, T‐cell receptor signaling pathway, natural killer cell‐mediated cytotoxicity, leukocyte transendothelial migration, cytokine receptor interaction, chemokine signaling pathway, and JAK‐STAT stat signaling pathway (Figure 4G).

### 3.4 RS calculation and survival analysis

**Figure 5:**
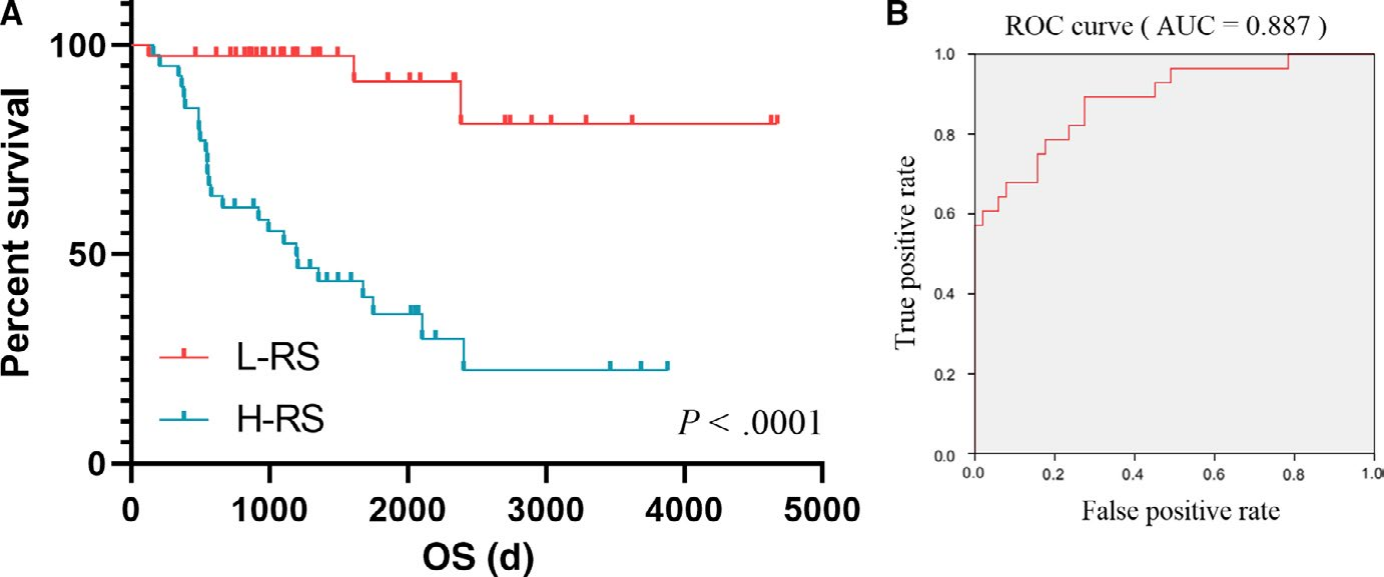
Prognostic value of RS. Survival analysis revealed that RS significantly related with poor survival outcomes (A, *P*＜ 0.0001). ROC curve indicated superior predictive accuracy in survival outcomes (AUC = 0.887) (B). AUC, area under the curve; ROC, receiver operating characteristic curve; RS, risk score

**Figure 6:**
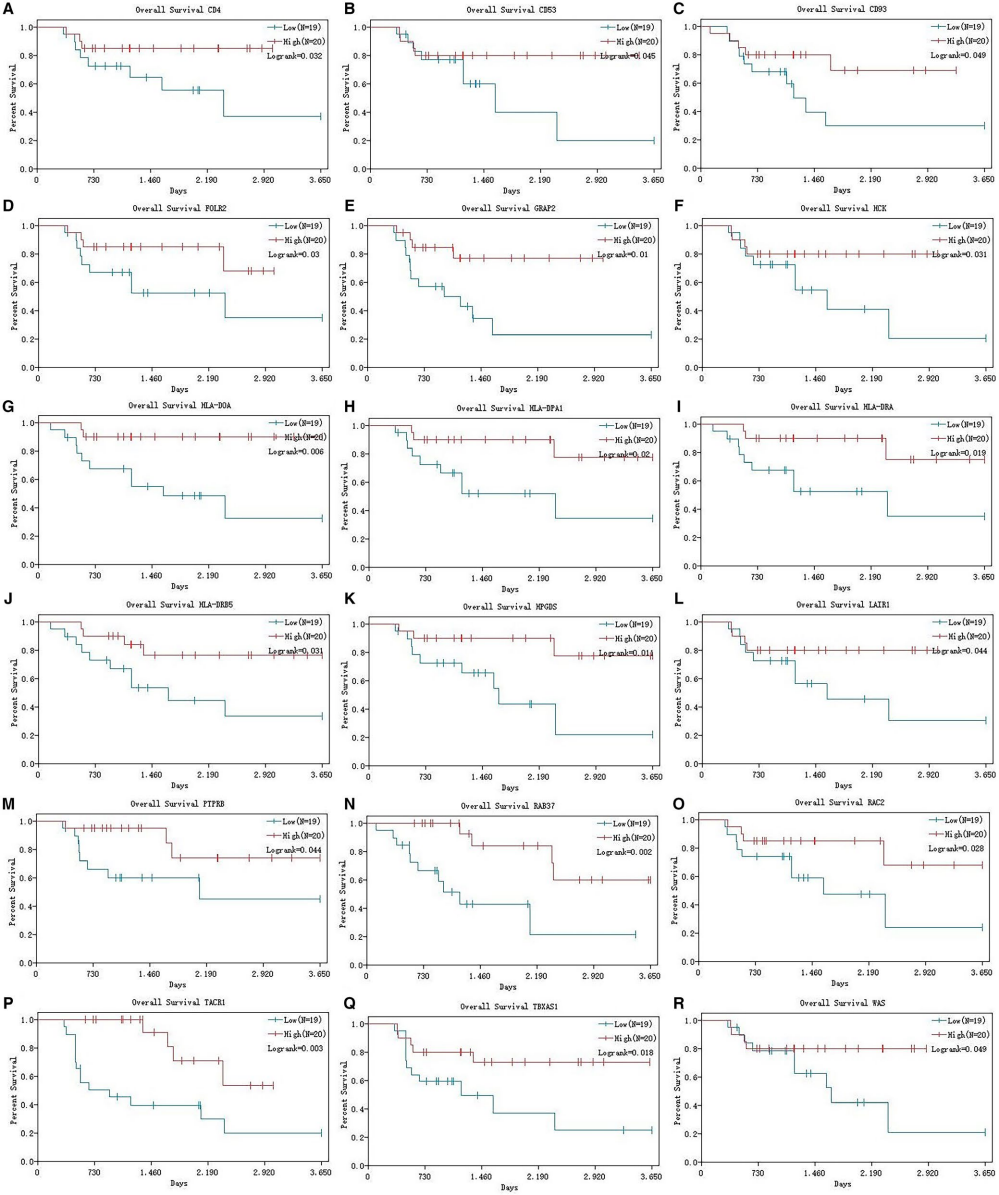
Survival analysis of hub genes. The K‐M curves revealed that 18 hub genes were significantly associated with survival outcomes

We calculated the risk score (RS) as: RS = −0.398*HLA‐DPA1‐0.656*RAC2‐0.247*HLA‐DRB5‐0.246*HPGDS + 0.985*PTPRB + 0.692*HCK + 0.092*HLA‐DOA + 0.005*RAB37 + 0.016*FOLR2‐0.538*GRAP2‐0.554*TACR1‐1.981*TBXAS1 + 0.102*HLA‐DRA + 1.802*CD53 + 1.545*LAIR1‐0.035*WAS‐0.645*CD93‐0.33*CD4 for all 79 ACC patients, and divided those patients into low‐RS group (n = 39) and high‐RS group (n = 40) (Table S4). Survival analysis revealed that high RS is significantly related with poor survival outcomes (Figure 5A, *P < *.0001). To further evaluate the prognostic value of RS, we performed the ROC curve and the area under the curve (AUC) was 0.887, which indicated superior predictive accuracy in survival outcomes (Figure 5B). The survival curves of 18 hub genes were exhibited in Figure 6A‐R.

### 3.5 Selected hub genes were associated with immune cell infiltration

**Figure 7:**
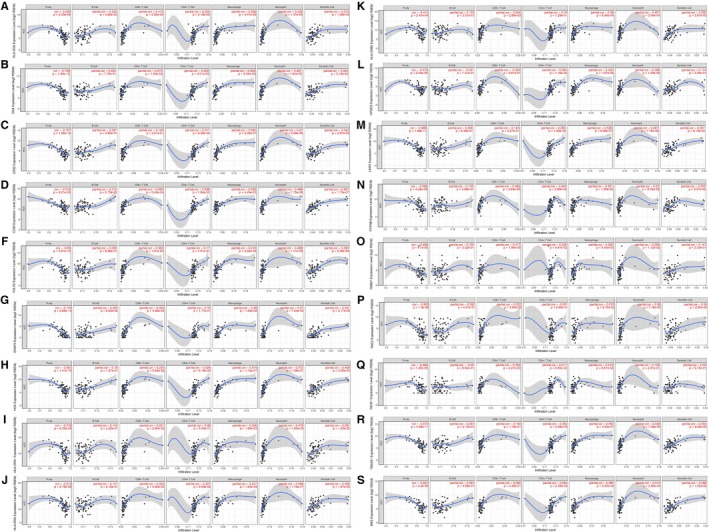
Selected hub genes were associated with immune cells infiltration. HLA‐DOA was significantly related with B cell, CD4 T cell, CD8 T cell, macrophage, neutrophil and dendritic cell infiltration (A). Other genes were related with some of the above immune cells infiltration (B‐S)

To further understand the relationship between hub genes and immune infiltration in ACC microenvironment, we explored the correlation ship in TIMER database. The results illuminated that HLA‐DOA was significantly related with B cell, CD4^＋^ T cell, CD8^＋^ T cell, macrophage, neutrophils, and dendritic cell infiltration (Figure 7A). Other genes were related to some of the above immune cells infiltration (Figure 7B‐S).

## 4 DISCUSSION

Recently, TME is getting increasing attention because of the profound research on the mechanism of immunotherapy and target therapy. Positive responses to immunotherapy usually depend on the interaction between tumor cells and immune regulation in TME. Previous studies had investigated the role of TME in various cancers. For example, Wood et al^32^ discussed the role of autocrine/paracrine signaling interactions, ECM remodeling, and cell‐cell interactions between tumor cells and the surrounding stroma, and the mechanisms could provide an important theoretical basis for target therapy in patients with NSCLC. Florent Petitprez et al^33^ demonstrated that the interactions between tumor cells and TME components were dependent on the original organ, treatment type, and oncogenic process. Unlike other studies our study identified specific signatures that associated with the infiltration of immune and stromal cells in ACC TME based on the transcriptional profiles in high‐quality datasets rather than focusing on the immune molecule and nontumor cells in TME.

In 2013, ESTIMATE was firstly introduced by Yoshihara et al^17^ to assess the immune cells infiltration and the presence of stromal cells based on gene expression data. This technique will help elucidate the role of TME to neoplastic cell and provide a new perspective on the occurrence of genomic alterations. In our study, immune scores, estimate scores, and stromal scores were calculated using ESTIMATE algorithm. Results showed that lower immune scores were significantly associated with DM and LR (*P* = .0204). Further K‐M analysis reflected that a lower immune score was closely related to poor OS (*P* = .0495), which was consistent with the previous research performed by Jia et al (GBM patients with lower immune scores had better OS than those with higher immune scores (442 d vs 394 d, *P* = .0537)).

PPI analysis was performed by Cytoscape software and SRING tool, from which 18 related hub genes were identified including CD4, CD53, CD93, FOLR2, GRAP2, HCK, HLA‐DOA, HLA‐DPA1, HLA‐DRA, HLA‐DRB5, HPGDS, LAIR1, PTPRB, RAB37, RAC2, TACR1, TBXAS1, WAS. Furthermore, potential pathways were obtained after GO‐enriched analysis such as T‐cell receptor signaling pathway, antigen processing and presentation of exogenous peptide antigen via MHC class II, T‐cell costimulation, MHC class II protein complex, and MHC class II receptor activity. Based on 18 selected hub genes which were closely related to ACC TME, a predictive model was developed. ACC patients with low RS had better survival outcomes than those with high RS (*P* < .0001). Additionally, satisfactory predictive efficiency (AUC = 0.887) revealed that our predictive model may have its potential for clinical application.

Protein tyrosine phosphatase receptor type B (PTPRB), also known as VE‐PTP, was regarded as an independent prognostic factor in patients with NSCLC. It was downregulated and was significantly associated with OS in NSCLC patients. Besides, PTPRB regulated the tumorigenesis and Scr phosphorylation.^34^ Weng et al^35^ reported that PTPRB promoted tumor metastasis and invasion of colorectal cancer via inducing epithelial mesenchymal transition. Obviously, it would be a new therapeutic target for treatment. Increasing evidence revealed that dysregulated Rab proteins were related to cancer progression.^36^, ^37^, ^38^ RAB37 was a metastasis suppressor by regulating exocytosis to the extracellular compartment, resulting in inhibition of cancer metastasis and neo‐angiogenesis.^39^, ^40^

In the analysis of the relationship between hub genes and immune infiltration, HLA‐DOA was identified to have significant correlation with B cell, CD4^＋^ T cell, CD8^＋^ T cell, macrophage, neutrophils, and dendritic cell infiltration. Previous researches had reported that infiltration of macrophage, CD4^＋^T cell, and CD8^＋^T cell played an important role in tumor prognosis. HLA‐DOA is a nonclassical class II MHC molecule. It exhibits very little sequence variation when compared with other classical HLA class II molecules. Studies on the role of HLA‐DOA in tumor prognosis are rare. Yukinori Okada^41^ reported that HLA‐DOA was an independent risk of anticitrullinated protein autoantibody‐positive rheumatoid arthritis.

Finally, some limitations should be taken into consideration in our research. First of all, lack of clinical data to validate the conclusions. We performed this study based on the public database via biological algorithm approaches and further clinical trials are needed before its application. Moreover, further investigations of these selected 18 TME‐related hub genes should be conducted, to have a better understanding of the regulatory mechanism in immune infiltrates, which may bring novel insights into the potential association of TME with ACC prognosis in a comprehensive manner.

## 5 CONCLUSIONS

In our study, we screened a list of TME‐related genes which predict poor survival outcomes in ACC patients from TCGA database. HLA‐DOA was significantly related with immune infiltration in ACC microenvironment. Further studies are needed to verify our conclusion.

## CONFLICTS OF INTEREST

The authors have no conflicts of interest to declare.
