# Supplementary figures and images for: Identification of prognostic genes in adrenocortical carcinoma microenvironment based on bioinformatic methods
Source: Cancer Med. 2019 Dec 19;9(3):1161–72. doi: 10.1002/cam4.2774 (PMC6997077; doi:10.1002/cam4.2774)

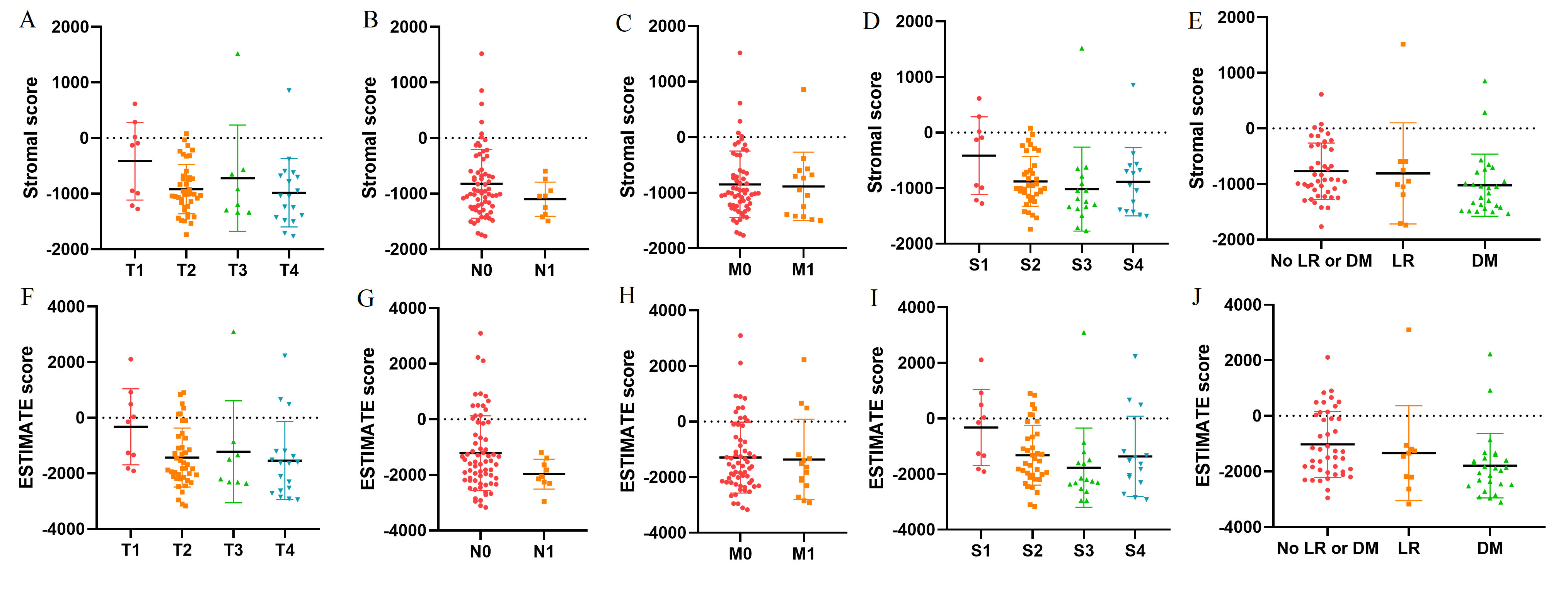

Supplement: Supplementary file 1 [file CAM4-9-1161-s001.png]

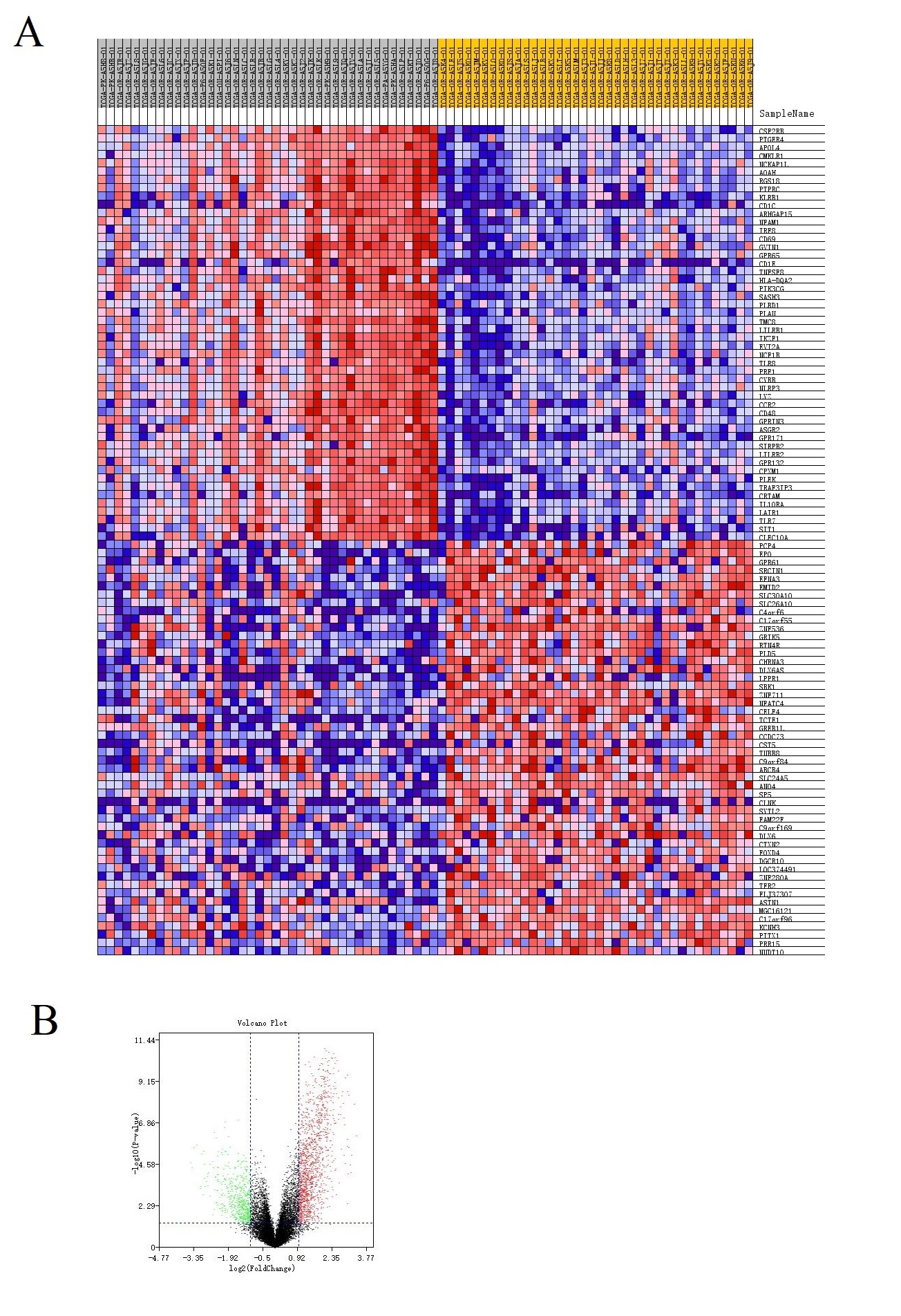

Supplement: Supplementary file 2 [file CAM4-9-1161-s002.png]
