# Supplementary material for: Identification of prognostic genes in adrenocortical carcinoma microenvironment based on bioinformatic methods
Source: Cancer Med. 2019 Dec 19;9(3):1161–72. doi: 10.1002/cam4.2774 (PMC6997077; doi:10.1002/cam4.2774)
Supplement: Supplementary file 3 [file CAM4-9-1161-s003.docx]

| ID | Stromal score | Immune score | ESTIMATE score |
| --- | --- | --- | --- |
| TCGA-OR-A5J1-01 | -1161.69 | -524.38 | -1686.07 |
| TCGA-OR-A5J2-01 | -569.12 | -765.96 | -1335.08 |
| TCGA-OR-A5J3-01 | -1295.48 | -1070.19 | -2365.67 |
| TCGA-OR-A5J5-01 | -1710.5 | -918.61 | -2629.12 |
| TCGA-OR-A5J6-01 | -730.82 | 64.28 | -666.54 |
| TCGA-OR-A5J7-01 | -1191.52 | -1013.02 | -2204.54 |
| TCGA-OR-A5J8-01 | 1519.45 | 1577.33 | 3096.78 |
| TCGA-OR-A5J9-01 | -1008.79 | -952.44 | -1961.24 |
| TCGA-OR-A5JA-01 | -1429.74 | -681.84 | -2111.58 |
| TCGA-OR-A5JB-01 | -677.06 | 1339.44 | 662.39 |
| TCGA-OR-A5JC-01 | -925.6 | -568.17 | -1493.76 |
| TCGA-OR-A5JD-01 | -828.23 | 99.35 | -728.89 |
| TCGA-OR-A5JE-01 | -949.16 | -386.98 | -1336.14 |
| TCGA-OR-A5JF-01 | -1032.35 | -1086.99 | -2119.35 |
| TCGA-OR-A5JG-01 | -949.32 | -690.02 | -1639.34 |
| TCGA-OR-A5JI-01 | -128.77 | -17.8 | -146.57 |
| TCGA-OR-A5JJ-01 | -1249.53 | -1052.41 | -2301.94 |
| TCGA-OR-A5JK-01 | -379.41 | 871.45 | 492.04 |
| TCGA-OR-A5JL-01 | -1273.69 | -545.2 | -1818.89 |
| TCGA-OR-A5JM-01 | -1502.61 | -1415.96 | -2918.57 |
| TCGA-OR-A5JO-01 | 615.06 | 1496.33 | 2111.39 |
| TCGA-OR-A5JP-01 | -834.61 | -1109.08 | -1943.69 |
| TCGA-OR-A5JQ-01 | -234.32 | 136.77 | -97.55 |
| TCGA-OR-A5JR-01 | -990.58 | -273.2 | -1263.78 |
| TCGA-OR-A5JS-01 | -1442.37 | -1031.13 | -2473.49 |
| TCGA-OR-A5JT-01 | -1140.87 | -694.11 | -1834.97 |
| TCGA-OR-A5JV-01 | -213.95 | 1118.14 | 904.19 |
| TCGA-OR-A5JW-01 | -1106.5 | -710.37 | -1816.87 |
| TCGA-OR-A5JX-01 | -914.29 | -577.16 | -1491.45 |
| TCGA-OR-A5JY-01 | -1044.77 | -342.78 | -1387.55 |
| TCGA-OR-A5JZ-01 | -956.54 | -361.74 | -1318.28 |
| TCGA-OR-A5K0-01 | -1535.32 | -914.7 | -2450.02 |
| TCGA-OR-A5K1-01 | -749.16 | -305.01 | -1054.17 |
| TCGA-OR-A5K2-01 | -1036.72 | -587.91 | -1624.62 |
| TCGA-OR-A5K3-01 | -1080.28 | -203.46 | -1283.74 |
| TCGA-OR-A5K4-01 | -1766.65 | -1181.25 | -2947.9 |
| TCGA-OR-A5K5-01 | -1334.86 | -976.97 | -2311.83 |
| TCGA-OR-A5K6-01 | -1009.58 | -440.21 | -1449.79 |
| TCGA-OR-A5K8-01 | -1242.14 | -769.03 | -2011.18 |
| TCGA-OR-A5K9-01 | -1051.28 | -1133.55 | -2184.83 |
| TCGA-OR-A5KO-01 | -1481.62 | -1365.58 | -2847.2 |
| TCGA-OR-A5KT-01 | 287.61 | 633.48 | 921.1 |
| TCGA-OR-A5KU-01 | -1014.36 | -1180.52 | -2194.88 |
| TCGA-OR-A5KV-01 | -1491.46 | -1466.95 | -2958.41 |
| TCGA-OR-A5KW-01 | -1041.5 | -1218.17 | -2259.67 |
| TCGA-OR-A5KX-01 | -1372.12 | -772.17 | -2144.29 |
| TCGA-OR-A5KY-01 | -596.34 | -596.95 | -1193.29 |
| TCGA-OR-A5KZ-01 | -595.45 | -660.33 | -1255.78 |
| TCGA-OR-A5L3-01 | -1216.03 | -694.1 | -1910.13 |
| TCGA-OR-A5L4-01 | -620.1 | -581.58 | -1201.68 |
| TCGA-OR-A5L5-01 | -91.49 | 128.04 | 36.55 |
| TCGA-OR-A5L6-01 | -926.22 | -225.37 | -1151.59 |
| TCGA-OR-A5L8-01 | -955.93 | -1378.17 | -2334.09 |
| TCGA-OR-A5L9-01 | -289.37 | 430.52 | 141.15 |
| TCGA-OR-A5LA-01 | -135.85 | 486.68 | 350.83 |
| TCGA-OR-A5LB-01 | -1389.25 | -1324.37 | -2713.62 |
| TCGA-OR-A5LC-01 | -702.35 | -812.08 | -1514.43 |
| TCGA-OR-A5LD-01 | -1239.48 | -1282.51 | -2521.98 |
| TCGA-OR-A5LE-01 | -1738.46 | -1432.98 | -3171.44 |
| TCGA-OR-A5LG-01 | -649.31 | -213.84 | -863.14 |
| TCGA-OR-A5LH-01 | -1223.99 | -390.02 | -1614.01 |
| TCGA-OR-A5LJ-01 | -1418.21 | -630.76 | -2048.97 |
| TCGA-OR-A5LK-01 | -326.3 | 1164.5 | 838.19 |
| TCGA-OR-A5LL-01 | -1067.84 | -811.91 | -1879.75 |
| TCGA-OR-A5LM-01 | -1296.08 | -739.73 | -2035.81 |
| TCGA-OR-A5LN-01 | -713.05 | 612.52 | -100.53 |
| TCGA-OR-A5LO-01 | -1485.54 | -1618.38 | -3103.92 |
| TCGA-OR-A5LP-01 | 79.52 | 47.54 | 127.05 |
| TCGA-OR-A5LR-01 | -680.35 | -407.13 | -1087.48 |
| TCGA-OR-A5LS-01 | -1420.37 | -1249.32 | -2669.69 |
| TCGA-OR-A5LT-01 | -1335 | -981.58 | -2316.58 |
| TCGA-OU-A5PI-01 | -734.76 | -1086.47 | -1821.23 |
| TCGA-P6-A5OF-01 | -783.65 | -809.81 | -1593.46 |
| TCGA-P6-A5OG-01 | 853.72 | 1378 | 2231.72 |
| TCGA-PA-A5YG-01 | -30.63 | 532.08 | 501.45 |
| TCGA-PK-A5H8-01 | -1005.09 | -523.91 | -1529.01 |
| TCGA-PK-A5H9-01 | -318.44 | -235.33 | -553.77 |
| TCGA-PK-A5HA-01 | 17.6 | 474.57 | 492.17 |
| TCGA-PK-A5HB-01 | -992.93 | -861.54 | -1854.48 |
